# Energy Absorption Characteristics of Polygonal Bio-Inspired Honeycomb Column Thin-Walled Structure under Quasi-Static Uniaxial Compression Loading

**DOI:** 10.3390/biomimetics7040201

**Published:** 2022-11-17

**Authors:** Shijie Wang, Hongxiang Xia, Yancheng Liu

**Affiliations:** 1 College of Civil and Architectural Engineering, Heilongjiang Institute of Technology, Harbin 150050, China; 2 School of Civil Engineering, Northeast Forestry University, Harbin 150040, China; 3 Longjian Road and Bridge Company Limited, Harbin 150009, China

**Keywords:** honeycomb structure, metals and alloy, structural impact, energy absorption, computer simulations

## Abstract

In this paper, we investigated the internal structure of the beetle elytra, i.e., two different structural forms I and II of the bio-inspired honeycomb column thin-walled structures (BHTS) that give the honeycomb sandwich structure frequently used in construction projects better mechanical properties and lightweight performance. BHTS specimens were fabricated by additive manufacturing selective laser melting (AM-SLM) using AlSi10Mg Al-Si alloy. In order to understand the effect of section angle number on BHTS during loading, quasi-static uniaxial compression tests were carried out and verified by numerical simulation. The experimental results showed that in the quasi-static uniaxial compression tests, the number of section angles greatly improved the energy absorption (EA) index of each BHTS: the average value of initial peak crushing force (PCF) of hexagonal BHTS increased by 108.82% and 43.44%, respectively, compared to triangular and rectangular BHTS. The average value of the mean crushing force (MCF) increased by 74.87% and 45.48%, respectively. The average value of EA increased by 89.02% and 46.64%, respectively. The results indicate that the number of section angles can be used as an effective way to enhance the EA of BHTS. This work can provide a reference for the design of high-efficiency energy absorbers and will be widely used in EA scenarios such as construction, transportation, etc.

## 1. Introduction

Thin-walled structures offer the advantages of high mechanical properties and light weight [1,2]. In recent years, thin-walled structures have been found to have good crashworthiness during collapsing [3,4]; therefore, they are widely used in civil construction, transportation, aerospace, and other industries for EA and protection devices. It has been shown that the cross-sectional structure is one of the key factors affecting the EA performance of thin-walled structures [5,6]. The crashworthiness of thin-walled structures with different cross-sectional configurations, including circular [7,8], square [9,10], pentagon [11], tapered tubes [12,13], honeycomb [14,15], multi-cell [16,17], and crisscross tubes [18] has been systematically investigated in detail. In these studies, the most severe plastic deformation occurred near the section angle of the tube, through the membrane deformation and bending deformation along the bending hinge line, in which a lot of energy can be consumed. For example, Ali et al. [11] conducted theoretical, numerical, and experimental studies on the dynamic axial crushing of pentagonal and cross-shaped tubes. They found that the energy absorbed by aluminum and steel pentagonal and crossed tubes was 60% and 92% higher than that of rectangular tubes in the same mass, respectively. Wu et al. [19] found that the number of cells in the cross-section can significantly determine the EA capacity. It can be seen that increasing the section angle and the number of cells in the section is an important way to enhance the EA of the structure from the study above.

**Figure 1:**
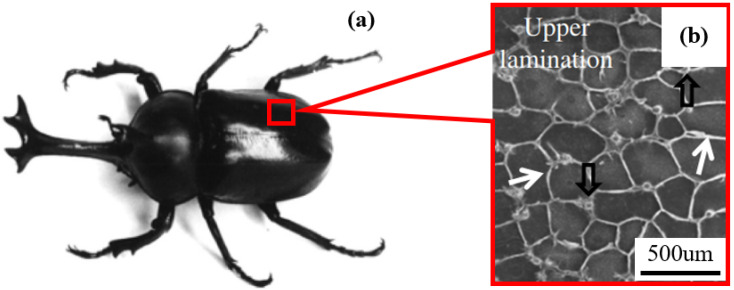
BHTS and its application: (**a**) the beetle *Allomyrina dichotoma*; (**b**) micromorphology of the honeycomb structure with columns [22] Copyright©5430520399371.

Important ways to enhance structural EA, such as increasing the section angle and the number of cells within the cross-section, are also reflected in many biological structures. Inspired by the biological inspiration of nature, many scholars have designed some hierarchical structures to enhance EA capacity. Hu et al. [20] developed a bionic honeycomb tubular nested structure (BHTNS) inspired by the micro-architecture of bamboo vascular bundles. The results showed that BHTNS had excellent specific energy absorption (SEA) and provided a reference for the design of efficient energy absorbers. Inspired by the internal structure of ladybugs, Xiang and Du [21] proposed a novel honeycomb structure BHTS by filling the column in a different way. The results showed that the EA characteristics of the bionic structure were better than those of the bionic honeycomb structure (BHS), which is a conventional honeycomb structure without columns. This honeycomb structure is expected to be used as an alternative structure in automobiles to improve crashworthiness. Hao and Du [22] proposed three bionic honeycomb thin-walled structures based on the internal structure of beetle elytra (Figure 1). The results showed that the SEA of each designed BHTS was greater than that of BHS and showed that BHTS represents an effective improvement in the enhanced EA capacity of honeycomb structures and potential applications in the field of protective equipment.

However, the design of the bionic honeycomb structure of the above investigation is complex, and the production often encounters difficult technical problems that have certain manufacturing difficulties using traditional methods. In recent years, additive manufacturing technology (AM) has become an excellent choice for manufacturing cellular structures with precise porosity and pore size due to its advantages of high precision in manufacturing parts and high material utilization [23,24], which stimulate the research of cellular structures in the AM area [25,26,27]. Xia et al. [28] used the AM-SLM technique to fabricate BHTS for uniaxial compression tests. Ngoc San Ha et al. [29] used fused deposition modeling (FDM) and fabricated a new bio-inspired hierarchical circular honeycomb (BHCH) for uniaxial compression tests. Good results were achieved by the experimental structures and numerical simulation, indicating the high feasibility of AM technology in manufacturing complex cellular structures.

In this study, the three BHTS structures proposed by Hao and Du, which mimic the internal hollow column structure of beetle elytra, were made polygon extensions to propose a novel structural design of triangular, rectangular BHTS. Motivated by these promising findings, the effect of the section angle on the EA of the BHTS was discussed through a series of low-velocity compression experiments and numerical simulations based on the internal structural features of the beetle elytra in previous work [28]. Section 2 briefly describes the new multi-deformation expansion design, the loading scheme with BHTS, and the corresponding numerical simulations. Then, Section 3 presents the results of quasi-static uniaxial compression experiments to obtain the EA behavior of BHTS under axial crushing conditions, and the effect of section angle on the EA performance of BHTS is discussed and verified by experimental results. Finally, some useful conclusions are drawn in Section 4. The results of this study can provide some references for the design of similar structures.

## 2. Materials and Methods

### 2.1. Specimen Design and Loading Method

#### 2.1.1. Tensile Test

**Figure 2:**
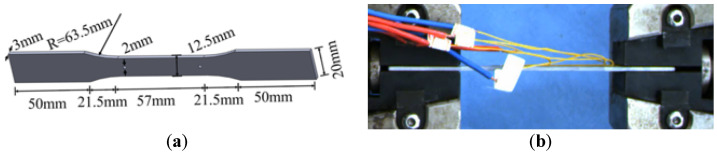
Specimen size and loading: (**a**) specimen size; (**b**) loading process.

Al-Si alloys have low density, high specific strength, good casting performance, and many excellent mechanical properties and are widely used in aerospace and automotive applications [30,31]. In order to determine the mechanical properties of BHTS material (AM-SLM AlSi10Mg), take the mechanical tests of porous structures fabricated through FDM by Ngoc San Ha et al. [29] for reference to test. The constitutive relation of nylon material was determined by a uniaxial tensile test, and numerical simulation was carried out using the obtained test data. The results showed that the finite element model (FEM) could effectively reflect the EA characteristics. According to the test concepts of the above scholars, the uniaxial tensile test was carried out. Five tensile dog-bone rod specimens were designed according to the ASTM E8/E8M standard [31], 200 mm long, 20 mm wide at both ends, 12.5 mm wide in the middle, with a circular arc diameter of 63.5 mm, and a 2 mm diameter hole in the middle (Figure 2a). By using SLM technology with high part-making accuracy and material utilization, each specimen was produced by an industrial-grade iSLM-280 professional metal printer. The tensile test was carried out on a Sanso Vertical 50 kN UTM5504GD universal testing machine with a controlled tensile speed of 0.2 mm/s (Figure 2b). Each sample was cleaned with ethanol before loading to remove dust and other potential contamination on its surface.

#### 2.1.2. Specimen Parameters and Numbering

**Figure 3:**
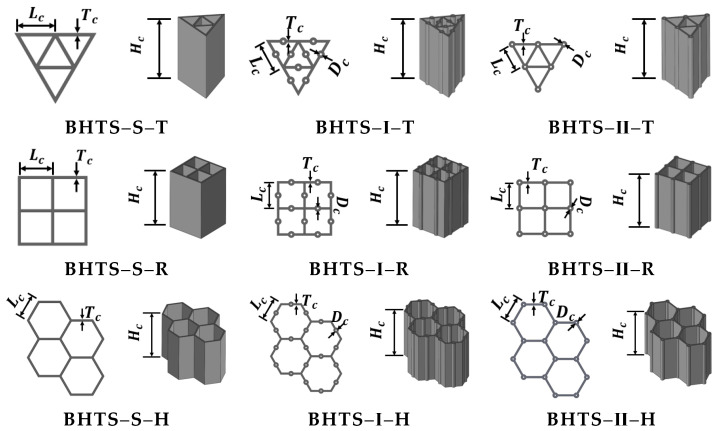
BHTS models.

The printed thin-walled structures models (Figure 3) were available in three configurations: triangular (T), rectangular (R), and hexagonal (H). The thickness $T_{c}$ of the manufactured thin-walled structure was 0.5 mm uniformly. The height $H_{c}$ of all specimens was 20 mm. The diameter $D_{c}$ of the circular thin-walled column structure was 0.5 mm. The side length $L_{c}$ of all polygons was 6 mm. The design of BHTS was based on the thin-walled honeycomb structure with columns of the BHS, and BHTS–S (BHS) was the control group. The BHTS–I and BHTS–II hollow columns were added to the center of the honeycomb wall and the wall connection, respectively. For simplicity, the manufactured thin-walled structures with triangular cross-sections were named as BHTS–S–T, BHTS–Ⅰ–T, BHTS–Ⅱ–T. By analogy, rectangles and hexagons were denoted as BHTS–S–R, BHTS–Ⅰ–R, BHTS–Ⅱ–R; BHTS–S–H, BHTS–Ⅰ–H, BHTS–Ⅱ–H, respectively, as shown in Table 1.

**Figure 4:**
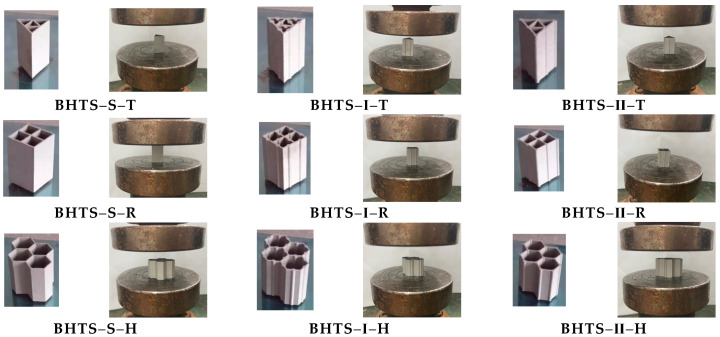
BHTS loading.

Figure 4 shows the design models made by AM technology. The compression load was applied with a constant speed of 0.02 mm/s by using the Sanso Vertical 50 kN UTM5504GD universal testing machine. All nine groups of specimens were crushed to 75% of their original length at the end of the compression tests, and each group contained two relatively close valid data.

### 2.2. Finite Element Models

#### 2.2.1. Modeling Process

The explicit finite element commercial software LS-DYNA [32] was used for the out-of-plane crush loading of BHTS. The AlSi10Mg material was simulated by the Cowper–Symonds isotropic hardening models. Considering the strain rate effect [33,34], the model expression is:

(1) $$\sigma_{Y}=\left[1+{\left(\frac{\dot{\varepsilon }}{C}\right)}^{\frac{1}{P}}\right]\left(\sigma_{0}+\beta E_{P}\varepsilon_{P}^{eff}\right)$$

In Equation (1), $\sigma_{0}$ is the initial yield stress; $\dot{\varepsilon }$ is the strain rate; β is the hardening coefficient; C and P are Cowper–Symonds strain rate parameters; $\varepsilon_{P}^{eff}$ is the effective plastic strain and, $E_{P}$ is the plastic hardening modulus.

**Figure 5:**
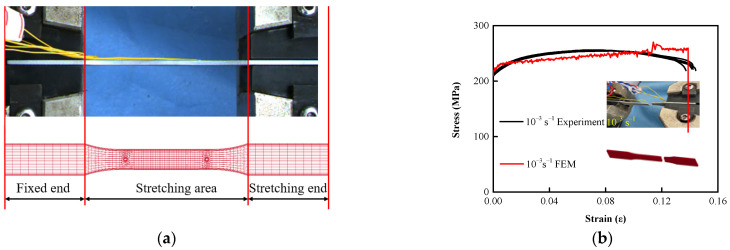
FEM: (**a**) boundary setting of the FEM; (**b**) comparison of the results.

In order to simulate the tensile process of AlSi10Mg tensile specimens, a FEM for low-velocity tensile analysis was established, as shown in Figure 5. Using coarse grids at the fixture end (tensile end and fixed end; mesh size was 5 mm), and finer grids were used in the fracture zone (mesh size was 1.25 mm) and transition zone (mesh size was 2 mm) to cope with larger plastic deformation in the finite element simulation (Figure 5a). The free translational degrees of the nodes on the contact surface between the fixed-end dog-bone rod and the fixture in three directions were constrained. The parameters in the FEM were adjusted according to the data collected from the tensile tests to be close to the test values and feedback (Figure 5b). It can be seen from the tests that the yield stress of the material fluctuated slightly at the level of 220 MPa, and other material characteristics from the experiments are shown in Table 2.

#### 2.2.2. Uniaxial Compression Test Models

**Figure 6:**
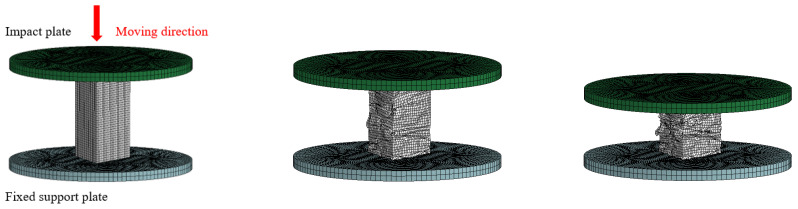

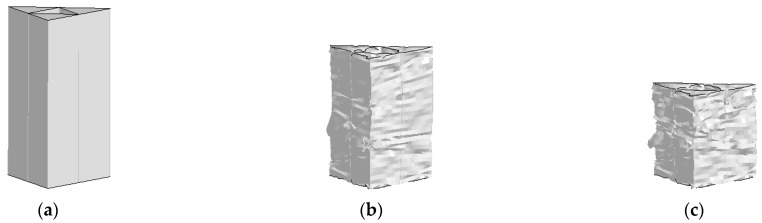
Schematic diagram of the compression process: (**a**) Compression start; (**b**) Slight compression; (**c**) Severe compression.

In the BHTS uniaxial compression tests, taking the loading process of BHTS–S–T as an example (Figure 6), the FEM consists of three parts: the fixed support rigid plate, the upper impact rigid plate, and the BHTS in between. The material of BHTS is *MAT_PLASTIC_KINEMATIC. The upper impact plate and the lower support plate are defined as rigid bodies by *MAT_RIGID. The rigid plate element was meshed by a constant stress body element, and the side length of the mesh was 1 mm. In the calculation, *DEFINE_CURVE was used to set the motion path, and *BOUNDARY_PRESCRIBED_MOTION_SET was used to define a time-varying linear displacement for the upper impact plate to ensure its uniform motion. The upper rigid plate constrained all displacements except the impact direction, and the lower rigid plate constrained all displacements. The complete integral Belytschko-Tsay [35,36] membrane element was used in the honeycomb aluminum tube. The honeycomb aluminum tube adopted a quadrilateral grid with a side length of 0.5 mm. Honeycomb structure model self-contact used single-sided automatic contact algorithm *CONTACT_AUTOMATIC_SINGLE_SURFACE honeycomb structure and support plate surface contact type selected point surface automatic contact algorithm *CONTACT_AUTOMATIC_NODES_TO_SURFACE [37], and the same algorithm was used for the contact type between the honeycomb structure and the impact plate.

### 2.3. Image Acquisition System

**Figure 7:**
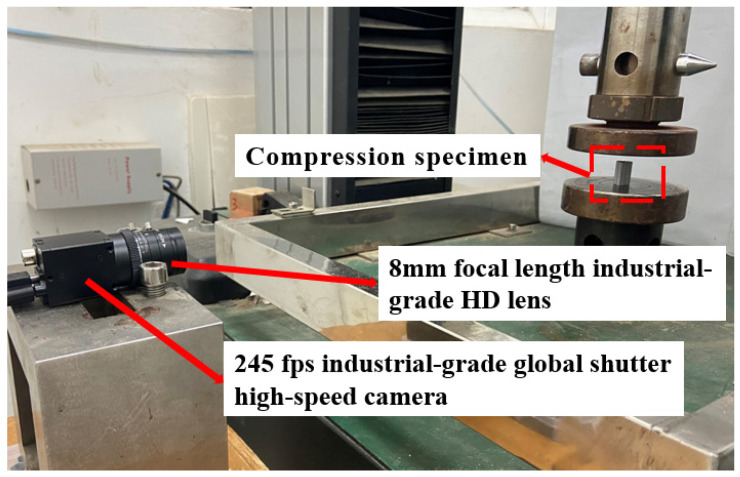
Site layout diagram.

In order to capture the complete deformation process of BHTS in each test for a more comprehensive understanding of the EA characteristics of BHTS under quasi-static compression, an industrial-grade global shutter high-speed camera MV-SUA133GC-T with a maximum frame rate of 245, equipped with an industrial-grade HD lens MV-LD-8-4M-G with a focal length of 8 mm was used to record the deformation process of the complete structure (Figure 7).

## 3. Results and Discussion

### 3.1. Experimental Phenomena

**Figure 8:**
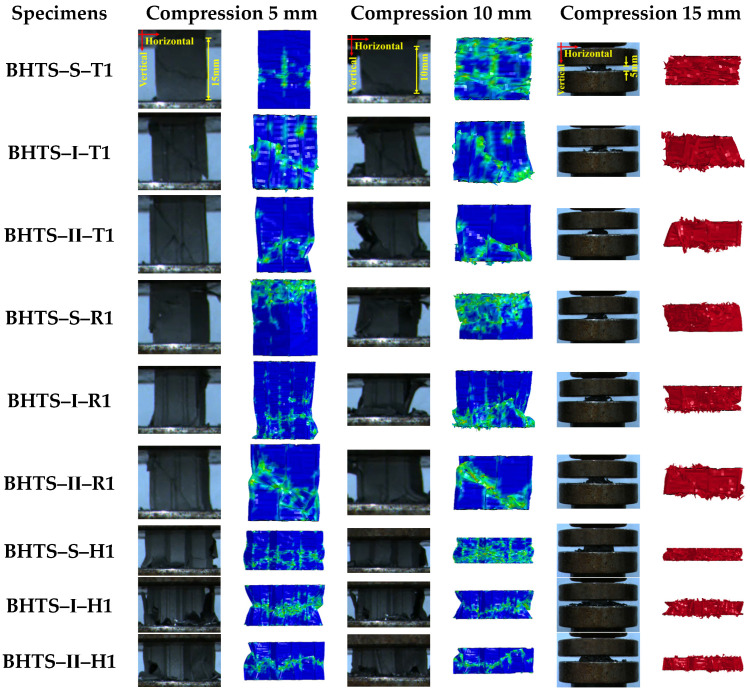
The crushing process of BHTS specimens under quasi-static uniaxial compression loading.

Two panels of close valid data were used for each sample. The classification explanation of each panel corresponding to each group is shown in Table 3. Figure 8 shows the loading process of BHTS under quasi-static uniaxial compression. The quasi-static out-of-plane uniaxial compression behavior of BHTS was analyzed. With the increase of compression displacement, the BHTS–S–T1 specimen was in the elastic deformation stage, and the middle began to expand. At the same time, the lateral deformation of the specimen showed regularities. As the displacement continued to increase, the specimen was in the elastic nonlinear deformation stage, and the lateral deformation increased greatly. Meanwhile, local bending buckling occurred on both sides of the specimen ribs. As displacement continued to increase, the specimen began to show more obvious shear failure followed by collapsing. The lower middle and upper of the specimen reached the failure strain and gradually crushed into the folded compact stage. In the folded and broken cycle, the specimen was finally compressed into a small broken piece. With the increase of compression displacement, the ribs and the cell walls on both sides of BHTS–I–T1 buckled at the same time and local bending buckling occurred, accompanied by a more obvious torsional response, then the local torsional-flexural buckling led to the collapse of the specimen. The ribs of BHTS–II–T1 buckled on both sides at the same time. After buckling, both sides of the ribs collapsed after obvious symmetrical shear deformation. BHTS–S–R1 had obvious local bending buckling at the top during compression. As the displacement increased, the top collapsed after shear failure. In BHTS–I–R1, with the loading of displacement, local torsional buckling occurred earlier, followed by local torsional–flexural buckling, and then collapsed after shear failure. BHTS–Ⅱ–R1 showed local buckling at the top with the increase of displacement and collapsed after a more obvious shear failure with the increase of displacement. BHTS–S–H1 collapsed after local bending buckling at the bottom with increased displacement, followed by single-celled shear failure. With the increase of displacement, local torsional–flexural buckling occurred in the middle and bottom of BHTS–I–H1. As the displacement continued to increase, single-celled and continuous shear failure occurred and then collapsed. With the increase of displacement, the middle and bottom of the BHTS–Ⅱ–H1 local bending buckling occurred, and with the continued increase of displacement, a single-celled with a continuous form of shear failure collapsed in the BHTS–Ⅱ–H1 later.

### 3.2. Typical Impact Force-Displacement Response Curves

**Figure 9:**
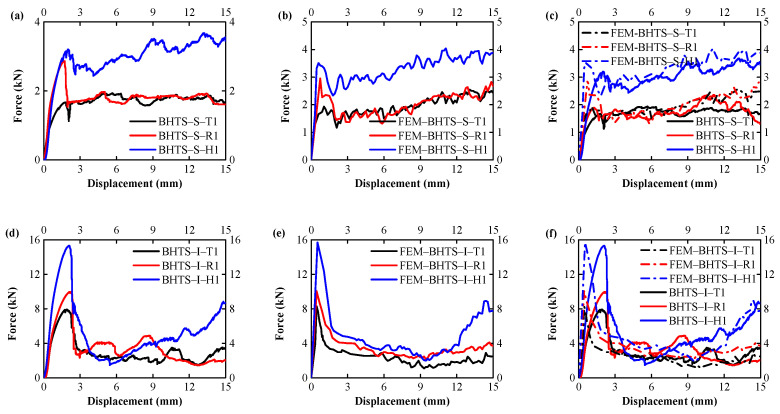

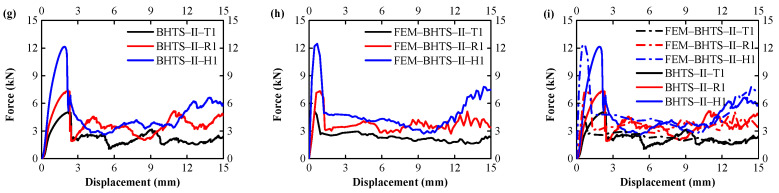
Force-displacement diagram of each structure under quasi-static uniaxial compression loading: (**a**–**c**) BHTS–S; (**d**–**f**) BHTS–Ⅰ; (**g**–**i**) BHTS–Ⅱ.

The force-displacement curves are used to characterize the mechanical and EA properties of the thin-walled structure, as shown in Figure 9. It can be seen that under dynamic impact loading conditions, the crushing force and EA have four different stages: elastic stage, plastic deformation stage, fracture stage, and densification stage. Taking BHTS–S–T1 as an example, it had a linear elastic deformation at 0–0.32 mm during loading. After exceeding the proportional limitation, the sample had obvious nonlinear elastic deformation at 0.32–1.86 mm due to the inevitable lateral displacement during loading. Linear elastic deformation and nonlinear elastic deformation are collectively referred to as the elastic deformation stage, where the crushing force increases rapidly to reach the initial PCF. The curve has no hardening stage; when the loading displacement reached 1.86 mm, local bending buckling and shear failure occurred and then collapsed. This stage is called plastic deformation and fracture, in which the crushing force decreases sharply. Entering the folding phase, the initial folding occurred after the displacement loading at 2.07 mm, and the specimen folded up and became densified into the progressive failure mode of strength failure and buckling coexisting. The strength curve rose at densification, decreased after buckling and strength failure, and repeatedly cycled until all compression was completed, which is called the densification stage. The progressive collapse mode phase after structural buckling was accompanied by plastic deformation and fracture. At this stage, the crushing force fluctuated periodically, and the crushing displacement was longer than the elastic and yielding stages. In addition, this stage achieved most of the EA. Finally, with the saturation of the fragment cracked, the structure was affected by the densification, and the crushing force of the force-displacement curve was on the rise.

**Figure 10:**
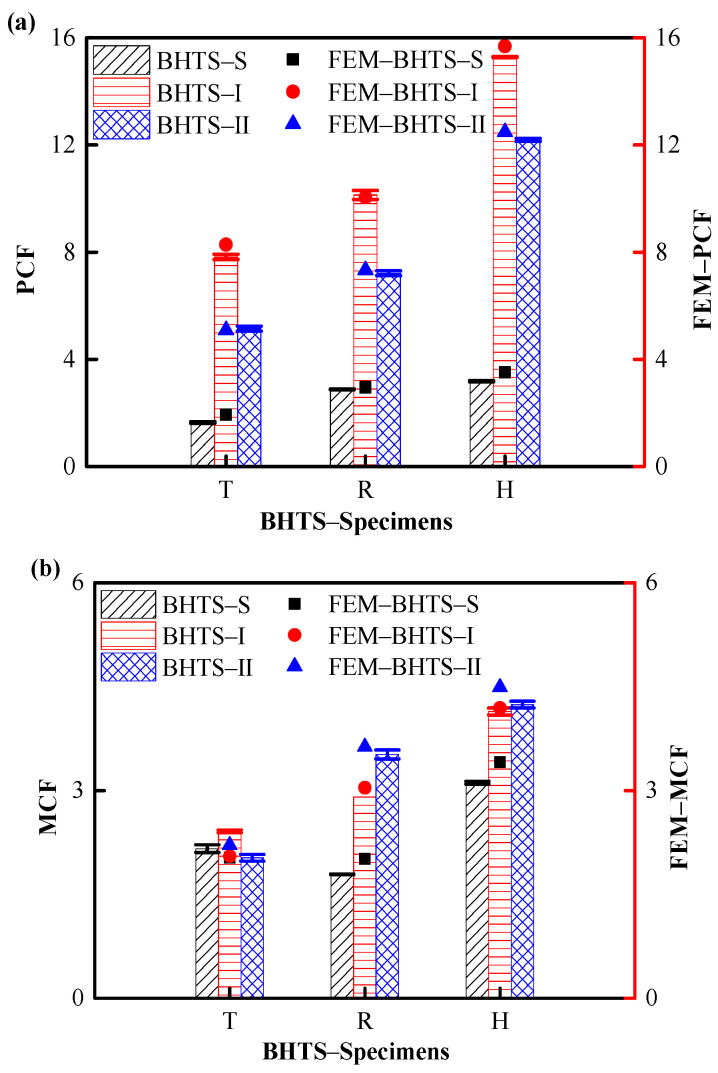
Statistics of PCF and MCF corresponding to different section angles: (**a**) PCF; (**b**) MCF.

Figure 10a,b compares the average PCF with MCF and the finite element simulation results of BHTS–S–T, BHTS–I–T, and BHTS–II–T specimens. It can be seen that the average PCF of all specimens increased with the increase of the section angle. Among the PCF of the three BHTS structures (S/I/II) in BHTS–H was 93.90%, 95.40%, 137.16%, and 10.42%, 50.84%, 69.07% larger than those of BHTS–T and BHTS–R, respectively. In the MCF analysis, except that BHTS–S–R was slightly smaller than BHTS–S–T, the average MCF of all specimens also increased with the increase of the number of section angles. Among the three BHTS structures (S/I/II), the PCF distribution of BHTS–Specimens–H was 43.98%, 71.78%, 108.87%, and 73.74%, 42.27%, 20.45% larger than that of BHTS–Specimens–T and BHTS–Specimens–R.

In addition, the experimental results were not much different from the numerical simulation results. Under quasi-static compression, the experimental mean values of PCF and MCF of triangle, rectangle, and hexagon were compared with the numerical results, and the experimental errors of PCF were 8.17%, 1.66%, and 5.12%, respectively. The test errors of MCF were 9.94%, 6.62%, and 5.58%, respectively, and the error mainly came from the inevitable lateral displacement during the loading process. It can be seen that the numerical simulation results are in good agreement with the experimental data, and the EA behavior of BHTS under impact loading was well simulated. It can be concluded that the present numerical models, including the adopted material models and the corresponding numerical algorithms, can effectively analyze the mechanical behavior of BHTS under quasi-static uniaxial compressive loading.

### 3.3. Energy Absorption Indexes

In this study, all crushing parameters were calculated directly from the force-displacement curve. PCF [38] is defined as the first peak load. MCF [39] was calculated as the average crushing force over a displacement range of 3 mm to 15 mm. The formula for calculating MCF is:

(2) $$MCF=\frac{1}{b-a}\int_{a}^{b}Fd\delta$$

In Equation (2), a is 3 mm and b is 15 mm, wherein, dδ is the compression displacement increment at the beginning of the densification state, and F is the compression load.

EA capacity is an important index to characterize the performance of porous materials. The mechanical properties of porous materials require compression shock absorption and EA. In order to study the EA performance of different configurations, two typical indicators were used as evaluation criteria: crushing force efficiency (CFE) [38,40] and SEA [41]. The EA indexes of different types of BHTS under different low-strain rates were evaluated. *CFE* is defined as the ratio of *MCF* to *PCF*, as shown in Equation (3):

(3) $$CFE=\frac{MCF}{PCF}$$

EA [42] is the energy dissipated during the crushing process of the specimens. According to Equation (3), the area under the force-displacement curve is determined and calculated based on the displacement from 0 to 15 mm.

(4) $$EA=\int_{0}^{b}Fd\delta$$

Due to the high strength-to-weight ratio of cellular structures, it is important to consider the energy absorbed per unit mass of the material. This is also known as SEA, which could be gotten by the dissipated EA divided by the specimen mass (m), as shown in Equation (5).

(5) $$SEA=\frac{EA}{m}$$

Generally speaking, the higher the SEA, the better the EA capacity of the BHTS is. In this paper, the commercial mathematical software Matlab [43] was used to compile the calculation program to summarize the data after calculation. Table 4 summarizes the PCF, MCF, CFE, EA, and SEA of all nine groups of samples. To facilitate comparison, we calculated the mean values of MCF, CFE, EA, and SEA for repeated samples.

### 3.4. Effect of Section Angle Number on EA in the Crushing Process

**Figure 11:**
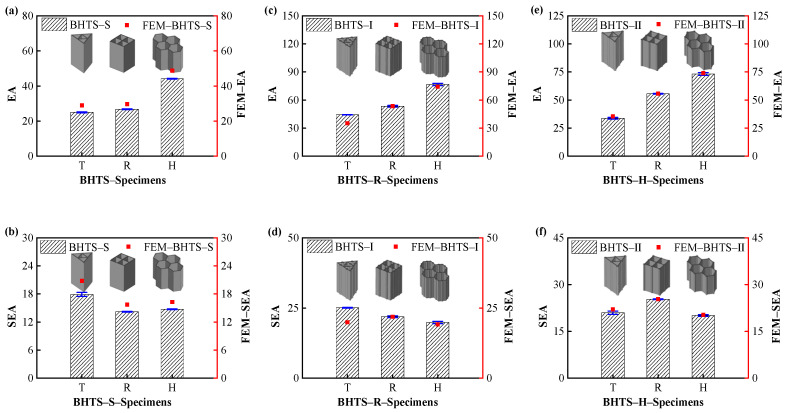
EA evaluation indexes of BHTS at different section angle number: (**a**,**c**,**e**) EA; (**b**,**d**,**f**) SEA.

The EA and SEA diagrams for each structure are shown in Figure 11. It can be seen that the EA of the designed BHTS rose with the increase of the section angle. In the BHTS–S–Specimens structure, the EA of BHTS–S–H was 76.80% and 64.79% larger than that of BHTS–S–T and BHTS–S–R, respectively. In the BHTS–Ⅰ–Specimens structure, the EA of BHTS–Ⅰ–H was 73.14% and 43.66% larger than that of BHTS–Ⅰ–T and BHTS–Ⅰ–R, respectively; In the BHTS–II–Specimens structure, the EA of BHTS–Ⅱ–H was 117.12% and 31.48% larger than that of BHTS–Ⅱ–T and BHTS–Ⅱ–R, respectively.

The SEA of the designed BHTS did not show a significant correlation with the section angle. The SEA of BHTS–S–H was −17.65% and 3.73% larger than that of BHTS–S–T and BHTS–S–R, respectively. The SEA of BHTS–S–H was −20.93% and −9.45% larger than that of BHTS–S–T and BHTS–S–R, respectively. The SEA of BHTS–S–H was −4.38% and −20.63% larger than that of BHTS–S–T and BHTS–S–R, respectively.

From the above analysis, it can be seen that the EA of the designed BHTS grew linearly with the increase in the number of section angles. In contrast, the SEA of BHTS did not have a significant correlation with the section angle, and there were two possible reasons for this phenomenon. (1) The honeycomb structure is convergent only after a certain number of cellular pores is reached [44]; in this paper, the number of four-cell elements was less according to the design that just meets the Chinese specification. (2) The size of the designed BHTS was small due to size effect [45]; more cell elements and larger sizes should be designed during the subsequent studies to find more accurate patterns.

### 3.5. Effect of Hollow Column Position on EA

**Figure 12:**
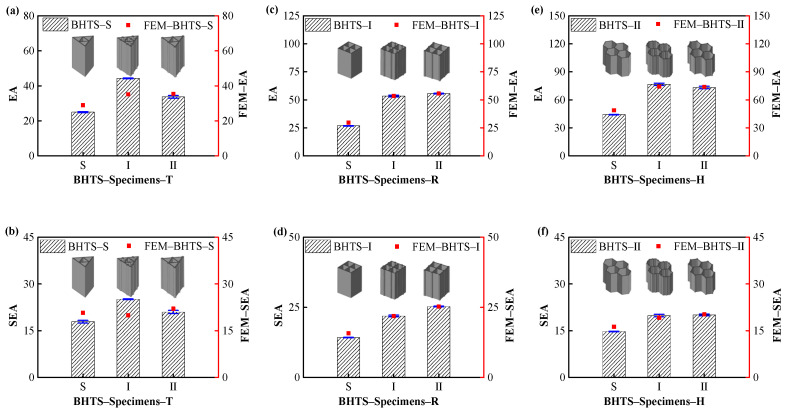
EA evaluation indexes of BHTS at different hollow column positions: (**a**,**c**,**e**) EA; (**b**,**d**,**f**) SEA.

The EA and SEA diagrams for each structure are shown in Figure 12. It can be seen that the EA of the designed BHTS shows a relatively obvious increasing trend with the installation position of the hollow column. In the BHTS–Specimens–T structure, the EA of BHTS–II–T was 77.32% and −23.85% larger than that of BHTS–S–T and BHTS–I–T, respectively. In the structure of BHTS–Specimens-R, the EA of BHTS–S–H was 99.17% and 4.33% larger than that of BHTS–S–R and BHTS–Ⅰ–R, respectively. In the structure of BHTS–I, the EA of BHTS–S–H was 99.17% and 4.33% larger than that of BHTS–S–T and BHTS–S–R, respectively.

The SEA of the designed BHTS also showed a significant upward relationship with the increase of the hollow column. In the BHTS–S–Specimens structure, the EA of BHTS–S–H was larger than that of BHTS–S–T and BHTS–S–R; 40.11% and 16.26%, respectively. The EA of BHTS–Ⅰ–H was 54.11% and 15.52% larger than those of BHTS–Ⅰ–T and BHTS–Ⅰ–R, respectively; The EA of BHTS–II–H was 34.53% and 1.26% larger than that of BHTS–II–T and BHTS–II–R, respectively.

From the analysis above, it is known that the EA of the designed BHTS increases with the increase of the hollow column. The EA of BHTS–I–Specimens and BHTS–II–Specimens was larger than that of BHTS–S–Specimens, and the EA of BHTS–II–Specimens was slightly larger than that of BHTS–I–Specimens. In the SEA analysis, the SEA of triangle BHTS–I–T was slightly smaller than that of BHTS–S–T. The SEAs of BHTS–I–Specimens and BHTS–II–Specimens in rectangle and hexagon were larger than that of BHTS–S–Specimens, and the SEA of BHTS–II–Specimens was slightly larger than that of BHTS–Ⅰ–Specimens. This indicates that the increase of cross-sectional columns and the distribution position can raise the EA and SEA of the honeycomb structure.

## 4. Conclusions

Due to the rapid development of materials science, the requirements for lightweight and high mechanical properties of building materials have grown sharply. Bio-inspired honeycomb column thin-walled structures (BHTS) is a new bionic structure proposed in recent years, with excellent mechanical and energy absorption properties. In order to study the effect of section angle number on the energy absorption of the proposed BHTS and ensure the accurate fabrication of complex honeycomb structures, two BHTS manufactured using AM-SLM technology were proposed. Through the combination of experiment and numerical simulation, the initial peak crushing force, mean crushing force, energy absorption, and specific energy absorption of the proposed structure were compared. The conclusions of this study are as follows:

- The bionic structure of the beetle elytra greatly enhances the energy absorption performance of the honeycomb structure. The design and installation location of BHTS hollow columns have a significant influence on the mechanical behavior of honeycomb structures.
- The initial peak crushing force, mean crushing force, and energy absorption of BHTS were greatly improved with the increase of section angle number (triangle, rectangle, and hexagon). The hexagonal structure has the best energy absorption indexes among the three structures, as described above.
- The finite element simulation results were in good agreement with the experimental results. This research method could provide an important reference for material designing, manufacturing, and modeling based on BHTS.
- More cell elements and larger sizes should be added to the subsequent design to make the test results obtained by convergence more accurate.

In general, it is proven that the number of section angles has a great influence on the energy absorption capacity of BHTS manufactured by AM-SLM AlSi10Mg. Considering that increase in the section angles has a significant enhancement effect on energy absorption significantly, BHTS has great potential in various engineering applications.

## Figures and Tables

**Table 1:** BHTS parameters of the study.

Specimen Label	Cell Side Length $L_{c}\;(\mathrm{mm})$	Cell Wall Thickness $T_{c}$ (mm)	Column Diameter $D_{c}$ (mm)	Column Height $H_{c}$ (mm)
BHTS–S–T	6.0	0.5	/	20.0
BHTS–Ⅰ–T	6.0	0.5	0.5	20.0
BHTS–Ⅱ–T	6.0	0.5	0.5	20.0
BHTS–S–R	6.0	0.5	/	20.0
BHTS–Ⅰ–R	6.0	0.5	0.5	20.0
BHTS–Ⅱ–R	6.0	0.5	0.5	20.0
BHTS–S–H	6.0	0.5	/	20.0
BHTS–Ⅰ–H	6.0	0.5	0.5	20.0
BHTS–Ⅱ–H	6.0	0.5	0.5	20.0

**Table 2:** Properties of Si-Al alloy AlSi10Mg.

Density	Young’s Modulus	Poisson’s Ratio	Initial Yield Strength
2670 kg/m^3^	69 ± 5 GPa	0.3	220 ± 10 MPa

**Table 3:** BHTS test set number.

Type	Group Number|Panel					
Specimen	1	BHTS–S–T1	4	BHTS–S–R1	7	BHTS–S–H1
FEM		FEM–BHTS–S–T		FEM-BHTS–S–R		FEM-BHTS–S–H
Specimen	2	BHTS–Ⅰ–T1	5	BHTS–Ⅰ–R1	8	BHTS–Ⅰ–H1
FEM		FEM-BHTS–Ⅰ–T		FEM-BHTS–Ⅰ–R		FEM-BHTS–Ⅰ–H
Specimen	3	BHTS–Ⅱ–T1	6	BHTS–Ⅱ–R1	9	BHTS–Ⅱ–H1
FEM		FEM–BHTS–Ⅱ–T		FEM-BHTS–Ⅱ–R		FEM-BHTS–Ⅱ–H

**Table 4:** Evaluation index values of mechanical properties of BHTS under different strain rates.

Group	Panel	Specimen	Mass	PCF	MCF	CFE	EA	SEA	Average Value				
Number									PCF	MCF	CFE	EA	SEA
			(g)	(kN)	(kN)	(%)	(J)	(J/g)	(kN)	(kN)	(%)	(J)	(J/g)
1	1	BHTS–S–T	1.38	1.66	2.21	133.29	25.28	18.32	1.64	2.16	131.87	24.96	17.90
	2		1.41	1.61	2.10	130.44	24.65	17.48					
2	1	BHTS–Ⅰ–T	1.78	7.91	2.43	30.64	44.41	24.95	7.82	2.41	30.84	44.26	25.08
	2		1.75	7.72	2.39	31.04	44.10	25.20					
3	1	BHTS–II–T	1.60	5.04	2.08	41.19	34.43	21.52	5.14	2.03	39.54	33.70	21.00
	2		1.61	5.23	1.98	37.88	32.97	20.48					
4	1	BHTS–S–R	1.89	2.86	1.79	62.50	27.01	14.29	2.88	1.79	61.96	26.78	14.21
	2		1.88	2.89	1.78	61.41	26.55	14.12					
5	1	BHTS–Ⅰ–R	2.43	9.96	2.91	29.30	52.50	21.60	10.13	2.91	28.76	53.34	21.90
	2		2.44	10.30	2.91	28.25	54.17	22.20					
6	1	BHTS–II–R	2.22	7.30	3.58	48.93	55.93	25.19	7.21	3.52	48.72	55.65	25.30
	2		2.18	7.12	3.45	48.50	55.37	25.40					
7	1	BHTS–S–H	3.02	3.20	3.13	97.81	44.29	14.67	3.18	3.11	97.96	44.13	14.74
	2		2.97	3.15	3.09	98.10	43.96	14.80					
8	1	BHTS–Ⅰ–H	3.85	15.30	4.19	27.39	77.89	20.23	15.28	4.14	27.10	76.63	19.83
	2		3.88	15.26	4.09	26.81	75.37	19.43					
9	1	BHTS–II–H	3.63	12.13	4.19	34.50	71.84	19.79	12.19	4.24	34.78	73.17	20.08
	2		3.66	12.24	4.29	35.06	74.50	20.36					

Note: The number of the panel represents the first or second valid test data.
